# Suppressing Emotions Impairs Subsequent Stroop Performance and Reduces Prefrontal Brain Activation

**DOI:** 10.1371/journal.pone.0060385

**Published:** 2013-04-02

**Authors:** Malte Friese, Julia Binder, Roger Luechinger, Peter Boesiger, Björn Rasch

**Affiliations:** 1 Department of Psychology, Saarland University, Saarbruecken, Germany; 2 Department of Psychology, University of Zurich, Zurich, Switzerland; 3 Institute for Biomedical Engineering, Swiss Federal Institute of Technology Zurich and University of Zurich, Zurich, Switzerland; Ghent University, Belgium

## Abstract

Abundant behavioral evidence suggests that the ability to self-control is limited, and that any exertion of self-control will increase the likelihood of subsequent self-control failures. Here we investigated the neural correlates underlying the aftereffects of self-control on future control processes using functional magnetic resonance imaging (fMRI). An initial act of self-control (suppressing emotions) impaired subsequent performance in a second task requiring control (Stroop task). On the neural level, increased activity during emotion suppression was followed by a relative decrease in activity during the Stroop task in a cluster in the right lateral prefrontal cortex (PFC) including the dorsolateral prefrontal cortex (DLPFC), an area engaged in the effortful implementation of control. There was no reliable evidence for reduced activity in the medial frontal cortex (MFC) including the anterior cingulate cortex (ACC), which is involved in conflict detection processes and has previously also been implicated in self-control. Follow-up analyses showed that the detected cluster in the right lateral PFC and an area in the MFC were involved in both the emotion suppression task and the Stroop task, but only the cluster in the right lateral PFC showed reduced activation after emotion suppression during the Stroop task. Reduced activity in lateral prefrontal areas relevant for the implementation of control may be a critical consequence of prior self-control exertion if the respective areas are involved in both self-control tasks.

## Introduction

The ability to control one’s impulses, emotions, thoughts and action tendencies is crucial for living in line with personal standards and social norms. Failures in self-control contribute to many social problems such as obesity, drug use, smoking, alcoholism, or crime [1], [2]. It is therefore of great interest to understand the processes underlying self-control failures.

Self-control can be defined as the ability to interrupt and override dominant response tendencies and replace them with different responses that are in line with current goals, personal standards, and social norms [3]. Behavioral evidence suggests that the ability to repeatedly exert self-control over time may be limited [4], [5]. Similar results have been obtained for the recruitment of executive functions [6] that subserve the various control processes involved in self-control [7]. Akin to a muscle that is tired after demands and fails to reach its highest level of performance, self-control performance is impaired by preceding efforts at self-control, even if these efforts have occurred in a different behavioral domain. Thus, exerting self-control may lead to a state of exhaustion, depletion or reduced motivation to control, which augments the likelihood of subsequent self-control failures. For example, after initial acts of self-control such as the control of attention, thoughts, or emotions, individuals showed decrements in control in that they reacted more aggressively to a provocation, ate more of a tempting snack, engaged in riskier behavior, and performed more poorly on executive function tasks [6], [8]–[10]. Importantly, an initial exertion of self-control does not impair performance on any subsequent task, but particularly on those tasks requiring effort and control [11].

Despite abundant behavioral evidence for impairing aftereffects of initial exertions of self-control [12], little is known about the neural mechanisms underlying these aftereffects. Prior research has investigated the neural correlates of self-control performance in single self-control tasks, but few studies have examined brain activity in two consecutive tasks and – in particular – the aftereffects of brain activity during the first task on brain activity during the second self-control task. The present study aimed at filling this gap. Evidence for the neural correlates of the aftereffects of self-control exertion would be valuable as it could shed light on the processes associated with a use-dependent weakening of control processes and thereby contribute more generally to theory building on self-control and its failures.

Self-control efforts have consistently been associated with increased activity in lateral prefrontal as well as medial frontal areas [7], [13]–[15]. These brain regions have been associated with different subprocesses of self-control performance. The detection of conflicts and errors as well as the recruitment of cognitive control is supported by a region in the medial frontal cortex (MFC, the conflict-and-error-detection zone, including the dorsal part of the anterior cingulate cortex, ACC) [16]–[18] and has been considered an efficient and largely resource-independent process [19]. In contrast, the implementation of control is considered a resource-demanding process, which has been associated with lateral prefrontal areas, in particular with the dorsolateral prefrontal cortex (DLPFC) [19]–[21].

Relying on behavioral measures alone, it is difficult to disentangle which of these processes are impaired after initial self-control efforts: conflict detection, the implementation of cognitive control, both, or none of these. In behavioral studies, aftereffects of self-control primarily affected effortful, resource-demanding processes [11], [22]. Applied to the neural level, these findings suggest decreased activity after self-control exertion than after a task not requiring self-control in brain areas that are typically engaged in the effortful implementation of control (e.g., the DLPFC), but less so in brain areas engaged in presumably effortless processes such as conflict detection (e.g., the conflict-and-error-detection zone in the MFC) [16].

Despite its popularity in behavioral (social) psychological research [12], very few studies have directly investigated the neural underpinnings of aftereffects of self-control. In a study using electroencephalography (EEG), Inzlicht and colleagues [23] reported that controlling one’s emotions reduced the error-related negativity as a marker of ACC activity in a subsequent Stroop task as compared to control participants. This result indicated an impairment of the conflict-and-error-detection process and suggested that the conflict-and-error-detection process may not be as resource-independent as hypothesized on theoretical grounds [19]. Aftereffects of self-control on lateral prefrontal areas involved in the implementation of control were not examined in this study. In addition, because no brain activity was recorded during the first self-control task, it remains unknown whether or not the reduced ACC activity during the Stroop task was preceded by increased demands on the ACC during the preceding emotion suppression task.

In another study using functional magnetic resonance imaging (fMRI), Richeson et al. [24] observed stronger activity in the right DLPFC in white volunteers while viewing black versus white male faces, indicating stronger engagement of control processes for black as compared to white faces. Individual differences in right DLPFC activity during this task were taken as an indicator of self-regulatory demands participants would face during an interaction with a black individual. In a separate session, the degree of implicit racial bias [25] predicted the impairment of cognitive control as indicated by poorer performance in a Stroop task after a short interracial interaction. Remarkably, the degree of activity in the right DLPFC during the face-viewing task mediated this effect of implicit racial bias on Stroop task performance. While this study provides indirect support for the assumption of aftereffects of DLPFC activity on subsequent self-control performance, a direct comparison of brain activity during the face-viewing task (or the interracial interaction) and the Stroop task was not conducted.

**Figure 1:**
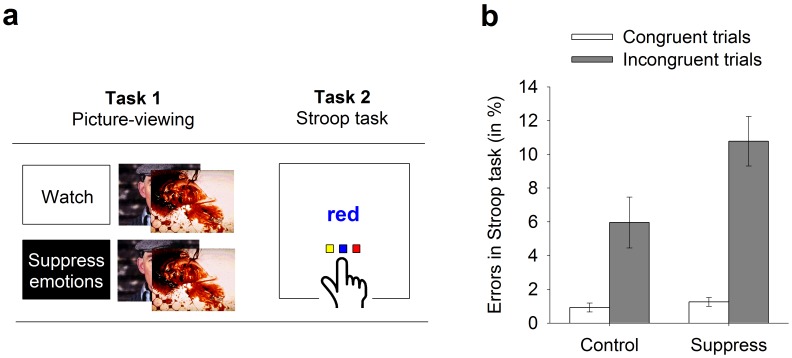
Overview of the experimental procedure and the behavioral results. (a) During the first task, the control group watched negative and neutral pictures. The suppression group suppressed emotions while watching the same pictures. Both groups subsequently completed a Stroop task. Brain imaging data was recorded during both tasks. (b) Participants in the emotion suppression group showed significantly stronger Stroop interference effects than participants in the control group. (Errors on incongruent minus errors on congruent trials.) Error bars represent standard errors of the mean (SE).

In the current study, we used fMRI to measure brain activity during both an initial (emotion suppression) and a subsequent self-control task (Stroop task, see Figure 1). Both suppressing emotions and inhibiting dominant responses during a Stroop task require increased activity in brain areas important for self-control [14], [26].

Applying the muscle metaphor to the level of brain activity we hypothesized that if a brain area involved in self-control is commonly activated in both the first (emotion suppression) and the second (Stroop) self-control task, then activity in this brain area would be reduced in the second task if both tasks are completed consecutively. This effect should be evident for brain areas involved in the resource-demanding, effortful implementation of control, such as the DLPFC [4], [19]. Hypotheses involving conflict detection in the medial frontal cortex including the ACC were less clear. Theoretical models expect conflict detection to be an efficient and resource-independent process [19], but previous research [23] suggests that it may in fact be sensitive to aftereffects of increased activity. To test these hypotheses, the right and left lateral PFC as well as the MFC served as regions of interest (ROI) and were functionally defined in an independent sample (see fMRI methods).

## Materials and Methods

### Ethics Statement

The study was approved by the ethics committee of the Canton of Zurich, Switzerland. Written informed consent was obtained from all participants.

### Participants and Design

Forty-two females naïve to the Stroop task participated in the study. We included only females because of the known gender-effects in emotional processing and related brain activity [27] such as differences in amygdala function during emotional experiences, memory for emotional events and different responsiveness to emotional material. We excluded one participant due to movements greater than 3 mm during the fMRI measurement, one because she did not follow instructions during the initial self-control task, and one due to self-reported claustrophobia interfering with adequate data acquisition in the narrow fMRI scanner. In the final sample, there were 20 participants in the suppression group and 19 participants in the control group. Mean age of the final sample was 23.54 years (*SD* = 2.84). Participants were randomly assigned to an emotion suppression group or a control group. In addition, we recruited an additional group of 19 volunteers (*M*_age_ = 23.25, *SD* = 3.54) to define our prefrontal regions of interest (see fMRI methods). All participants received CHF 25/hour (approximately US $23).

### Procedure

First, participants briefly practiced both the picture-viewing task and the Stroop task outside the fMRI scanner to become familiar with them. After practicing, participants were positioned in the fMRI scanner. Their head was fixated in the coil using small cushions, and they were told not to move their head. Next, they performed both tasks while functional MR-images were acquired followed by an anatomical scan. Outside the fMRI scanner, participants filled in a questionnaire including several control questions, the manipulation check, demographic data, and were debriefed (see Figure 1a).

### Materials and Measures

#### Picture-viewing task

Twenty-four neutral and 24 negative pictures were taken from the International Affective Picture System (1 = most negative/least arousing, 9 = most positive/most arousing) [28]. The sets differed significantly in valence (*M*_neg_ = 1.97, *SD*_neg_ *= *0.33, *M*_neu_ = 5.07, *SD*_neu_ = 0.23, *F*(1, 46) = 1409.25, *p*<.001). In addition, negative pictures were significantly more arousing than neutral pictures (*M*_neg_ = 5.85, *SD*_neg_ *= *0.76, *M*_neu_ = 3.08, *SD*_neu_ = 0.62, *F*(1, 46) = 190.99, *p*<.001). Pictures of the same valence were randomized within blocks of 4 pictures (6 neutral blocks, 6 negative blocks, 48 pictures in total). The order of the blocks was also randomized, except that two blocks of negatively valenced pictures always occurred at the end of the task. Prior to every picture block, a fixation-cross appeared on the screen for 500 ms followed by the instruction presented for 1.5 seconds (“suppress” [unterdrücken] or “watch” [anschauen] depending on condition). Each picture was presented for 7 seconds. The interblock-interval was 5 seconds. Similar to previous research [12], [29], participants in the suppression group were instructed to suppress all emotions elicited by the pictures and to control their facial reactions. In the control group, participants were told that it was okay to allow emotions in response to the pictures.

#### Stroop task

In each trial, a stimulus appeared in blue, red, or yellow ink and participants were instructed to react to the ink color and ignore the semantic meaning of the stimulus by pressing one of three keys. In (in)congruent trials, the semantic meaning of the word did (not) match the ink color. In neutral trials, ‘XXX’ appeared in one of the 3 colors. There were 120 congruent, 30 neutral, and 30 incongruent trials that were presented in a fixed pseudo-random order. Stimuli remained on the screen until the participant gave a response or until 1500 ms had passed followed by a fixation cross. Each trial was 2200 ms long. There were 6 congruent trials immediately prior to and 6 congruent trials immediately after the 180 critical trials. These 12 additional trials were discarded. Errors on incongruent versus congruent trials as well as incongruent versus neutral trials were analyzed as a function of experimental condition (suppression vs. control).

#### Arousal ratings

In the picture-viewing task, participants indicated their subjective arousal (large, medium, small) on a three-point scale by pressing a button on a button box with their dominant hand after each block. One participant failed to provide arousal ratings.

#### Manipulation check

Participants answered two questions indicative for exerted self-control on 7-point rating scales: ‘How exhausting was it for you to follow the instructions during the picture-viewing task?’ and ‘How much did you have to concentrate to follow the instructions during the picture-viewing task?’ (α = .94). The manipulation check was added later in the experiment. The respective data are therefore only available for the last 29 participants.

### fMRI Methods and Procedures

Measurements were performed on a Philips Achieva 1.5 T wholebody MR unit equipped with an eight-channel Philips SENSE head coil. Functional time series were acquired with a sensitivity encoded, single-shot echo-planar sequence (SENSE-sshEPI) sensitive to BOLD contrast (T2* fast field echo with the following acquisition parameters: TR (repetition time) = 3000 ms, TE (echo time) = 45 ms, FOV (field of view) = 22 cm, acquisition matrix = 80×80, interpolated to 128×128, voxel size: 2.75×2.75×4 mm^3^, 32 contiguous axial slices without gaps and SENSE acceleration factor R = 2.0). By using a midsagittal scout image, 32 contiguous axial slices were placed tilted by 20° to the anterior–posterior commissure plane covering the entire brain. The first two acquisitions were discarded to allow for T1 saturation. The picture-viewing task as well as the Stroop task consisted of 155 functional scans each. The tasks were measured in two runs, separated by a short 1-min break. A projector displayed stimuli on a screen in the scanner room, which subjects viewed through a mirror mounted on top of the head coil. For each subject, we also acquired high-resolution (0.86×1×2 mm, 55 slices, TE = 15 ms, 3 averages) T1-weighted anatomical images.

Preprocessing was performed using SPM5 (Statistical Parametric Mapping; Wellcome Department of Cognitive Neurology, London, U.K.; http://www.fil.ion.ucl.ac.uk/spm/) implemented in Matlab 2009a. Volumes were slice-time corrected to the first slice, realigned to the first acquired volume, normalized to each individual T1 image, and smoothed using a 8 mm full-width-at-half-maximum Gaussian kernel. A 128-sec-cutoff high-pass filter was added to the confound partition of the design matrix to account for low-frequency drifts, and a correction for intrinsic autocorrelations was included in the analysis.

### Statistical Analyses of the fMRI Data

All statistical analyses of fMRI data were performed using standard procedures provided by SPM5.

#### First level analysis

In the picture-viewing task, evoked hemodynamic responses to different stimulus categories (negative pictures, neutral pictures) were modeled for each subject with a box-car function corresponding to stimulus presentations convolved with a canonical hemodynamic response function within the context of a general linear model (GLM). In addition, the button presses during the arousal ratings were modeled as events using a delta (stick) function. Six movement parameters from spatial realigning were included as regressors of no interest.

In a separate GLM model for the Stroop task, event-related regressors were used to model the display of congruent words, incongruent words and neutral “XXX” trials using a delta (stick) function corresponding to stimulus presentations convolved with a canonical hemodynamic response function within the context of a GLM. Only correct trials for congruent words, incongruent words and “XXX” trials were used in the respective regressors. All error trials were modeled in a separate regressor of no interest, together with the first 6 and the last 6 congruent trials that were excluded on the behavioral level. In addition, six movement parameters from spatial realigning were included as regressors of no interest.

#### Second level analysis

In the two experimental groups (suppression vs. control), a random-effect group analysis was conducted on contrast images of the picture-viewing task (negative vs. neutral images) and the Stroop task (incongruent vs. congruent trials) from the individual analysis, comparing parameter estimates in the suppress group against the estimates in the control group. We used a factorial model with the two factors ‘type of task’ (picture-viewing vs. Stroop, within-subjects factor) and ‘experimental group’ (suppression vs. control, between-subjects factor) in SPM5. We calculated the interaction between the type of task (picture-viewing vs. Stroop) and the experimental group (suppression vs. control) using directional *t*-contrasts. The interaction was further analyzed using planned pair-wise contrasts between the two experimental groups in each task separately. In addition, we extracted the parameter estimates for each subject at the peak voxel to visualize the interaction.

**Figure 2:**
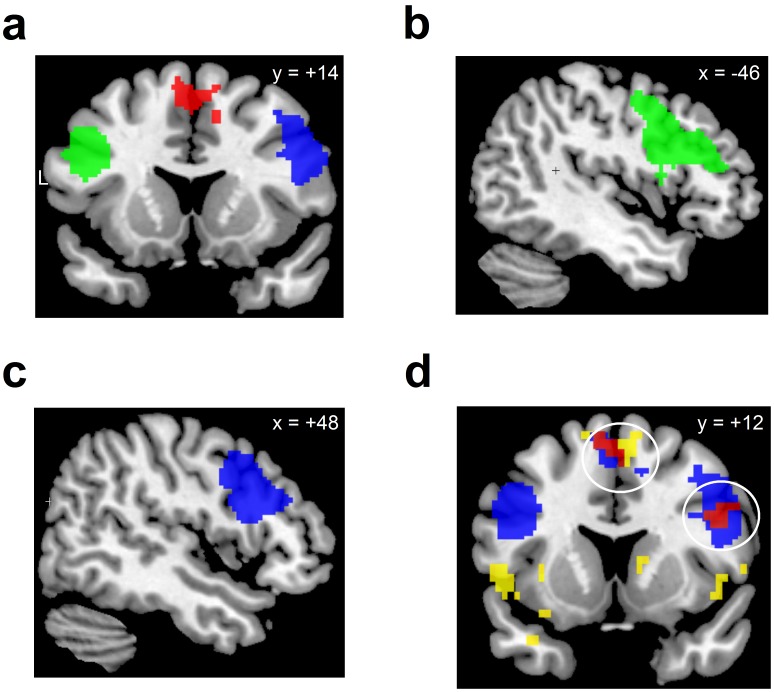
Regions of interest (ROI) used to identify effects of self-control exertion on prefrontal brain activity, superimposed on a coronal (a) or sagittal slice (b–c) of an anatomical template image. The three ROIs were functionally defined by the brain activation during the Stroop task (incongruent minus congruent trials; thresholded at *p = *.001, uncorrected, minimum cluster size k >28) in an independent sample of 19 healthy women who watched only neutral pictures before performing on the Stroop task. Blue: right lateral prefrontal cortex (lateral PFC); green: left lateral PFC; red: medial frontal cortex (MFC). (d) Overlap of activation during suppression of emotions during picture-viewing (yellow) with regions of interests obtained during the Stroop task (blue). Overlap is mainly observed in the right lateral prefrontal cortex and in the medial prefrontal cortex (red). Regions of activation are displayed with a minimum cluster size of k >28 at an individual voxel threshold of *p* = .001 in both analyses, superimposed on a coronal slice of an anatomical template image.

ROI definition and significance threshold: To functionally define regions of interest (ROIs) for the second-level analysis, we recruited an independent sample of 19 participants who followed the same procedure as the suppression and the control group with the exception that they watched 12 blocks of only neutral pictures before performing the identical Stroop task as participants in the main study. To protect against false-positive activations, we used a minimum cluster size as statistical threshold [30]. The nonarbitrary voxel cluster size was determined using the software AlphaSim [31]. On the basis of a Monte Carlo simulation (10000 iterations), clusters were considered significant at an overall whole brain false-positive rate of 5% when exceeding k >28 voxels at an individual voxel height threshold of *p* = .001. Based on the contrast “incongruent vs. congruent” trials in the Stroop task, we defined three regions of interest: The right lateral PFC (k = 279 voxels, peak activation at [47 17 32]; *Z* = 5.04), the left lateral PFC (k = 607 voxels, peak activation at [−41 33 20]; *Z* = 5.60; and the MFC (k = 129 voxel, peak activation at [14 19 56]; *Z* = 4.92, see Figure 2). The two lateral prefrontal regions included the following anatomical regions (as defined by the AAL toolbox for SPM5 [32]: 38%/16% (right/left, respectively) of the region in the inferior frontal gyrus (pars opercularis, BA 44), 24%/24% in the inferior frontal gyrus (pars triangularis, BA 45), 17%/19% in the middle frontal gyrus (BA 46, BA 9) and 16%/35% in the lateral precentral gyrus (BA 6). The MFC region was located to 79% in the anterior part of the supplementary motor area (pre-SMA; BA 6), 20% in the superior frontal and medial frontal gyrus as well as the mid-cingulate gyrus (BA 32). It thus encompassed large parts of the conflict-and-error-detection zone [16]. For significance testing, we combined the three regions to a single search mask (total number of voxels: 1015 voxels, see Figure 2a–c).

Within this search volume, we used a significance threshold of *p*<.05, corrected on the cluster level for multiple comparisons using the software AlphaSim (minimum cluster size of k >7 voxels at an individual voxel threshold of *p* = .001). For an exploratory whole brain analysis, we used the same significance threshold as in the ROI-definition group (minimum cluster size of k >28 voxels at an individual voxel threshold of *p* = .001, see above).

Locations are indicated in coordinates of the Montreal Neurological Institute (MNI). Anatomical labeling of clusters was based on the AAL toolbox for SPM5 [32]. For identification of Brodmann areas, we used the Talairch demon provided by Lancaster and Fox (www.talairach.org) [33]. Transformation of MNI to Talairach space was performed by a MATLAB tool (icbm_spm2tal.m) [34], [35] provided by the BrainMap Project at the Research Imaging Institute of the University of Texas Health Science Center San Antonio (brainmap.org).

### Correlational Analyses

We extracted the parameter estimates for each subject for the left and right lateral PFC as well as the medial prefrontal ROI in the Stroop task to calculate correlations with Stroop performance using SPSS. ROIs were defined by the independent ROI-definition group. For each ROI, the first eigenvariate of all voxels within the respective ROI was calculated. Parameter estimates were extracted for each subject for the contrast “congruent vs. incongruent trials”.

## Results

### Manipulation Check

As expected, participants in the suppression group reported that the picture viewing task was more exhausting for them and that they had to concentrate harder (*M*_suppress_ = 3.63, *SD* = 1.99) than participants in the control group (*M*_control_ = 1.57, *SD* = 0.62; *t*(27) = 3.72, *p = *.001, Cohen’s *d* = 1.40). Conversely, participants in the suppression group subjectively rated the emotional pictures as less arousing (*M*_suppress_ = 2.01, *SD* = 0.58) than participants in the control group (*M*_control_ = 2.89, *SD* = 0.14; *t*(36) = 6.53, *p<*.001, Cohen’s *d* = 2.09).

### Main Behavioral Results

Participants’ average response latency explained significant variance in several analyses involving the Stroop task. We therefore controlled for this variable in all analyses involving error rates in the Stroop task (both behavioral and fMRI analyses). All reported means are estimated marginal means controlling for average response latency.

Consistent with prior findings, exertion of self-control in the picture-viewing task (i.e. by suppressing one’s emotions) impaired the subsequent performance in the Stroop task. Participants in the suppression group had higher error rates in incongruent trials (*M*_suppress_ = 11.15, *SE* = 1.35) as compared to participants in the control group (*M*_control_ = 5.57, *SE* = 1.38; *F*(1, 36) = 8.31, *p = *.007, partial η^2^ = .187). No difference was observed for congruent trials (*M*_suppress_ = 1.22, *SE* = 0.25, *M*_control_ = 0.96, *SE* = 0.26; *F*(1, 36) = 0.54, *p = *.466, partial η^2^ = .015). The interaction between the factors ‘group’ (suppression vs. control) and ‘trial type’ (congruent vs. incongruent) was significant (mixed analysis of covariance [ANCOVA], *F*(1, 36) = 7.99, *p* = .008, partial η^2^ = .182; see Figure 1b). In addition, the analysis revealed the classic Stroop interference effect of more errors on incongruent than on congruent trials (*F*(1, 36) = 4.23, *p* = .047, partial η^2^ = .105). The main effect of group was also significant (*F*(1, 36) = 8.08, *p* = .007, partial η^2^ = .183).

There was no effect of prior emotion suppression on error rates in neutral trials in the Stroop task (*M*_suppress_ = 1.95, *SE* = 0.55, *M*_control_ = 2.71, *SE* = 0.57; *F*(1, 36) = 0.90, *p = *.349, partial η^2^ = .024). When neutral trials were included in the analysis in a 2 (group: suppression vs. control) ×3 (trial type: congruent vs. neutral vs. incongruent) mixed ANCOVA the group × trial type interaction remained significant (*F*(1.21, 43.71) = 7.61, *p* = .006, partial η^2^ = .175, Greenhouse-Geisser-corrected) as well as when analyzing only neutral and incongruent trials (*F*(1, 36) = 8.25, *p* = .007, partial η^2^ = .186). When additionally controlling for arousal ratings, the reported interactions between experimental group and trial type remained significant (all *p*s <.05) indicating that differences in error rates between experimental conditions were not due to differences in experienced arousal.

One could argue that error rates on incongruent trials were in fact not increased for the suppression group, but for some reason decreased for the control group as compared to normal circumstances. To rule out this possibility we compared the experimental groups with the independently recruited ROI definition group who had only seen neutral pictures before the Stroop task. Error rates were comparable for congruent trials across groups (*M*_control_ = 0.92, *SE* = 0.24, *M*_ROI_def_ = 0.77, *SE* = 0.24, *M*_suppression_ = 1.20, *SE* = 0.23), but the suppression group had higher error rates on incongruent trials (*M*_control_ = 5.97, *SE* = 1.29, *M*_ROI_def_ = 7.17, *SE* = 1.31, *M*_suppression_ = 11.46, *SE* = 1.27), resulting in a significant interaction between experimental group and trial type (*F*(2, 54) = 4.52, *p* = .015, partial η^2^ = .143). Thus, as expected, participants who had exerted self-control in the picture-viewing task showed impaired performance during the subsequent Stroop task during the difficult incongruent trials, but not during easier congruent and neutral trials.

Response latencies showed the regular Stroop interference effect between congruent and incongruent trials (*F*(1, 37) = 94.81, *p<*.001, partial η^2^ = .719), but no interaction with the experimental group (*F*(1, 37) <1, *p = *.584, partial η^2^ = .008). Stroop interference effects (incongruent-congruent) based on errors and based on latencies were reliably correlated (*r* = .50, *p* = .001).

### fMRI Data

According to the hypothesis, a brain area would be sensitive to aftereffects of self-control if it was *more* engaged by participants exerting self-control in the picture-viewing task (emotion suppression, contrast: negative vs. neutral pictures), but *less* engaged in the subsequent self-control task (Stroop, contrast: incongruent vs. congruent trials) relative to participants in the control group. Such a finding would suggest that participants who had previously exerted self-control were less able or less willing than participants in the control group to recruit the respective brain area in the Stroop task. Statistically, this hypothesis translates into an interaction between the type of task (picture-viewing vs. Stroop, within participants) and the experimental group (suppression vs. control, between participants). As region of interest (ROI), we used a functionally defined mask of prefrontal activation during the Stroop task (containing the left and right lateral PFC as well as the medial prefrontal cortex) obtained from the independent control group (see methods section “ROI definition”).

**Figure 3:**
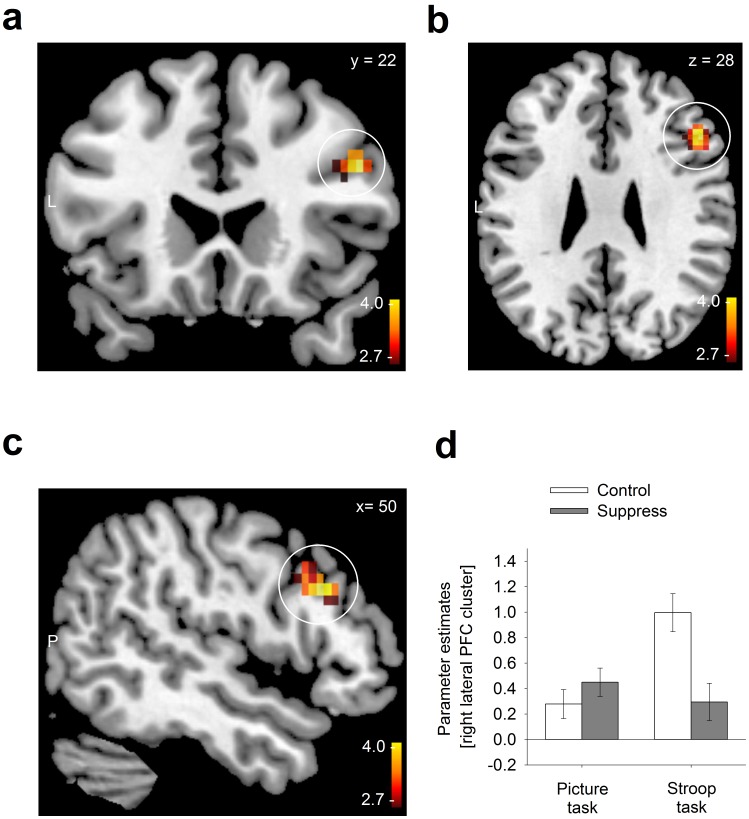
Aftereffects of self-control exertion on brain activity in the right lateral prefrontal cortex. (a–c) More activity in participants suppressing emotions during the picture-viewing task relative to control participants was followed by relatively less activity during the Stroop task in a cluster located in the right middle frontal gyrus (BA 9), extending into the inferior frontal gyrus (BA 9/45). *t*-values of the interaction contrast are color coded and displayed at a threshold of *p* = .005 (uncorrected) superimposed on a coronal (a), axial (b) and sagittal (c) slice of an anatomical template image. (d) Parameter estimates (arbitrary units) of the averaged activity of the right lateral PFC cluster for the experimental groups. Parameter estimates are extracted as first eigenvariate of all activated voxels in the cluster. Means ± SE are indicated.

When examining the contrast incongruent versus congruent trials in the Stroop task, we observed a trend for an interaction between type of task and experimental group within the search volume in the right lateral PFC ([50 22 28] (MNI coordinates), Z_max = _3.53, k = 6, *p* = 0.065, corrected for multiple comparison within the ROI). This trend was theoretically predicted, but failed to reach conventional limits of significance. However, using neutral trials (“xxx”) instead of congruent trials (i.e. employing the contrast ‘incongruent-neutral’), a similar and highly significant interaction was revealed in the exact same location in the right lateral PFC ([50 22 28]; Z_max = _3.63, k = 22, *p* = .004, see Figure 3 and Table 1). The more pronounced interaction effect in the analysis using neutral trials is possibly due to a more precise estimation of the BOLD response for neutral trials (mean inter-stimulus interval of the 30 neutral trials: 13.2 s) as compared to the much more frequently occurring congruent trials (mean inter-stimulus interval of the 120 congruent trials: 3.3 s). The peak activation of the interaction is located in the right middle frontal gyrus (BA 9, DLPFC), and the cluster extends to the right inferior frontal gyrus (BA 9/BA 45, see Figure 3a–c). As illustrated in Figure 3d, in the picture-viewing task, participants who were asked to suppress their emotions activated the right lateral PFC more strongly than the control group. By contrast, in the Stroop task, the suppression group failed to reach the same level of activation in the right lateral PFC as the control group did, overall resulting in a significant interaction. In the overall interaction analysis, no significant cluster was observed in the MFC or in the left lateral PFC. Controlling for possible differences in emotional arousal by adding arousal ratings as a covariate basically did not alter the results (e.g., interaction in the right lateral PFC, [50 22 28], Z_max_ = 3.65; k = 6, *p = *.065 for congruent trials; [50 22 28], Z_max_ = 3.93; k = 14, *p = *.014 for neutral trials). The exploratory whole brain analysis revealed no further significant clusters of activation in the interaction analysis (all uncorrected *p*s >.001 and k <29).

**Table 1:** Results within the functionally defined prefrontal search volume for the interaction between activity during the picture-viewing and the Stroop task for different types of analyses.

						MNI (mm)				
		BA		k_E_	*x*		*y*	*z*	*Z_max_*	*p*
**Analysis I: incongruent vs. congruent trials**										
*Picture-viewing: Suppression>control Stroop: Suppression<control*										
Middle/inferior frontal gyrus	R	9/45		6	*50*		*22*	*28*	3.53	.065
*Inverse contrast*	*No suprathreshold clusters*									
**Analysis II: incongruent vs. neutral trials**										
*Picture-viewing: Suppression>control Stroop: Suppression<control*										
Inferior/middle frontal gyrus	R		9/45	22	*50*		*22*	*28*	3.81	.004**
*Inverse contrast*	*No suprathreshold clusters*									

*Note.* BA: Brodmann area; R/L: right/left hemisphere; k_E_: number of voxels; *Z*_max_: Z-value at the peak activation of the cluster. Analysis thresholded at *p<*.001 (uncorrected) inside the prefrontal ROI. Cluster-level corrected *p*-values are indicated (**: *p*<.01). The search volume was functionally defined in an independent sample performing the Stroop task. It encompasses two activated clusters in the left and right lateral prefrontal cortices (including the inferior and middle frontal gyrus and the lateral precentral gyrus) as well as one activated cluster in the medial frontal cortex (supplementary motor area and superior frontal gyrus).

Our hypothesis that a brain area is sensitive to aftereffects of self-control relies on the assumption that there is an overlap between prefrontal brain areas activated during emotion suppression and during the Stroop task. We tested this assumption by inclusively masking the activation during the picture-viewing task of the suppression group with the prefrontal ROIs representing activation during the Stroop task in an independent sample. The analysis indeed revealed that the suppression group showed increased activation in the right lateral PFC ([47 8 28], Z_max_ = 4.04; k = 37, *p = *.001) and the MFC ([−8 14 60], Z_max_ = 4.17; k = 44, *p*<.001), which overlapped with the prefrontal activation in the Stroop task of the ROI-definition group (Figure 2d). The right lateral PFC activation was located in the inferior frontal gyrus (pars opercularis, BA 44, 57.6%; pars triangularis, BA 45, 27.5%) and overlapped with the observed interaction between task type and experimental group reported in the previous section. The activation in the medial prefrontal cortex was located in the bilateral supplementary motor area (BA 6) in the conflict-and-error-detection zone [16]. In a similar analysis involving activity during the picture-viewing task of the control group not suppressing emotions instead of the suppression group, no overlapping activation between the picture-viewing task and the Stroop task was observed. Thus, in spite of overlapping activation between emotion suppression and Stroop performance in both the medial frontal cortex as well as the right lateral prefrontal cortex, only the right lateral prefrontal cortex was sensitive for aftereffects of prior self-control exertion as revealed by the reported significant interaction between task type and experimental group.

When analyzing the difference between experimental groups in each task separately, the whole brain analysis of the picture-viewing task revealed a significant cluster of activation in the medial frontal gyrus/pre-SMA, which was more strongly activated in the suppression group as compared to the control group (see Table 2). Two further clusters in the right lateral PFC as well as one in the left lateral PFC were more strongly activated in the suppression as compared to the control group at a more lenient threshold. All three of these clusters were located in the middle frontal gyrus (BA 9: [36 41 32], *Z*_max_ = 4.07; k = 13, [44 19 40], *Z*_max_ = 3.66; k = 6; and [−41 47 16], *Z*_max_ = 3.58; k = 6; all *p*s <.001, uncorrected). However, because these clusters did not pass our predefined cluster threshold for the whole brain analysis, we do not further consider these results. In the Stroop task, no suprathreshold clusters were observed for the “congruent vs. incongruent” contrast. For the “neutral (“xxx”) vs. incongruent” contrast, one cluster in the left occipital lobe and one cluster in the precuneus were significantly less activated in the suppression group than in the control group (see Table 2).

**Table 2:** Results of the exploratory whole brain analysis for each task separately.

					MNI (mm)				
		BA	k_E_	*x*		*y*	*z*	*Z_max_*	*p*
**Analysis IAPS task: negative vs. neutral pictures**									
*Suppression>control*									
Medial frontal gyrus/pre-SMA		6/8	39	*0*		*25*	*44*	3.98	.013*
*Inverse contrast*	*No suprathreshold clusters*								
**Analysis Stroop I: incongruent vs. congruent trials**									
*Suppression>control*			*No suprathreshold clusters*						
*Inverse contrast*	*No suprathreshold clusters*								
**Analysis Stroop II: incongruent vs. neutral trials**									
*Suppression>control*			*No suprathreshold clusters*						
*Inverse contrast*									
Occipital lobe		17	37	−*19*		−*83*	*4*	4.35	.016*
Precuneus		30	31	*8*		−*52*	*16*	4.35	.034*

*Note.* BA: Brodmann area; R/L: right/left hemisphere; k_E_: number of voxels; *Z*_max_: Z-value at the peak activation of the cluster. Analysis thresholded at *p<*.001 (uncorrected) in a minimum of k_E_ >28 adjacent voxels. Cluster-level corrected *p*-values are indicated (*: *p*<.05).

### Correlational Analyses

On an exploratory basis, we calculated bivariate correlations between Stroop performance and mean brain activity extracted from the three ROIs during the Stroop task separately for each experimental group (see Table 3). We did not have a priori hypotheses about the strength and direction of these correlations, but we considered these data sufficiently interesting and potentially useful for readers to present them here.

**Table 3:** Bivariate correlations between Stroop interference effects and mean brain activity in the three regions of interest (ROI) during the Stroop task.

	Right lateral PFC ROI	Left lateral FPC ROI	MFC ROI
Stroop interference in suppression group	−.59* (*p* = .009)	−.51* (*p* = .020)	−.29 (*p* = .208)
Stroop interference in control group	.28 (*p* = .253)	.55* (*p* = .016)	.25 (*p* = .304)

*Note*. *: significant after Bonferroni-Holm correction for multiple comparisons [36]. Stroop interference effects are calculated as errors on incongruent trials minus errors on congruent trials, residualized by average response latency. Brain activity during the Stroop task is calculated as the difference between incongruent vs. congruent trials. We extracted the parameter estimates for each subject for the left and right lateral PFC as well as the medial prefrontal ROI as first eigenvariate of all voxels in the respective ROI.

Correlations differed remarkably between experimental groups. In the suppression group, more activity in the left and right lateral PFC was significantly associated with better Stroop performance (i.e. smaller Stroop interference effects; *r*_rLPFC_ = -.59, *p* = .009; *r*_lLPFC_ = -.51, *p* = .020). The direction of the relationship was the same for the MFC ROI, but this correlation was not significant (*r*_MFC_ = -.29, *p* = .208). By contrast, in the control group greater activity was associated with greater Stroop interference effects, and this relationship was significant for the left lateral PFC ROI (*r*_lLPFC_ = .55, *p* = .016). The direction of the relationship was the same for the right lateral PFC ROI and the MFC ROI, but these correlations were not significant (*r*_rLPFC_ = .28, *p* = .253; *r*_MFC_ = .25, *p* = .304). All correlations with *p*<.05 remained significant after correction for multiple comparisons based on the Bonferroni-Holm procedure [36]. The difference in correlations between the suppression and the control group was significant for the right and left lateral PFC ROIs (*z*_rLPFC = _2.66, *p* = .008; *z*_lLPFC = _3.39, *p*<.001, all analyses two-tailed). Running these analyses based on the contrast “neutral vs. incongruent trials” (for both Stroop performance and parameter estimates) revealed a similar pattern of correlations.

## Discussion

Our data provides evidence for a role of the right lateral PFC in the aftereffects of self-control on subsequent control attempts. Participants who had recruited the right lateral PFC while suppressing their emotions in the picture-viewing task committed more errors and showed less activity in the same area during a subsequent attempt at self-control in the Stroop task relative to participants in a control condition who had recruited this area not as strongly during the first task. In addition, two brain areas were particularly strongly involved during both the emotion suppression task (as indicated by the suppression group) and the Stroop task (as indicated by an independent sample). One of these areas was in the right lateral PFC including the cluster showing the above-described pattern of strong recruitment in the emotion suppression task and relatively reduced subsequent activation during the Stroop task. The second overlapping area was located in the MFC, but did not show a similar pattern of aftereffects of self-control exertion. Together, these results suggest that one way initial self-control efforts may impact on subsequent attempts at self-control is by impairing the later implementation of control through the right lateral PFC.

A large number of studies implicate the lateral PFC, particularly the DLPFC, in self-control activities such as inhibition, thought and emotion suppression [13], [14], [17], [21], [26]. Evidence for a causal role of the DLPFC in self-control is corroborated by studies temporarily compromising the efficient functioning of this brain area using low-frequency repetitive transcranial magnetic stimulation (rTMS) [37]. In these studies, the application of rTMS on the right DLPFC decreased the subsequent ability to control self-interested impulses in the context of economic game paradigms [38]. While low frequency rTMS produces a transient reduction in cortical excitability for several minutes, in the present study the activation of the right lateral PFC during self-control had functionally equivalent effects by distorting the efficient functioning of this brain area in a subsequent attempt at self-control.

In contrast to the right lateral PFC, we found no reliable evidence for a similar pattern of reduced activity in the MFC ROI after emotion suppression even though part of this ROI was strongly involved in the emotion suppression task and the Stroop task (as indicated by an independent sample). This suggests that in the present study the conflict-and-error-detection process was less affected by the repeated exertion of self-control during emotion suppression and the Stroop task than the right lateral PFC. This result is consistent with the theoretical assumption that the conflict-and-error-detection process is largely automatic and resource-independent [19], but inconsistent with an EEG study in which the error-related negativity as a marker for ACC activity in the MFC was reduced in a Stroop task after the exertion of self-control [23]. The present study considerably extends this latter study in that it measured brain activity during both the first *and* the subsequent self-control task, thereby allowing to test the more specific hypothesis of neural aftereffects of self-control exertion as a function of strong involvement of a brain area in both tasks. In addition, using fMRI we were able to identify the reduced activity in the right lateral PFC, an analysis less feasible with EEG as EEG has a much lower spatial resolution as compared to fMRI.

It is worth discussing the pattern of the observed interaction between the type of task (picture-viewing vs. Stroop) and experimental group (suppression vs. control) in some detail (cf. Figure 3). During the picture-viewing task, participants in the suppression group were explicitly instructed to exert control and suppress all emotions that would arise in response to the pictures they saw. It is therefore plausible that these participants recruited brain areas commonly involved in emotion suppression more strongly (e.g., the right lateral PFC) [14], [39], [40] than participants who saw the same pictures but were not instructed to suppress their emotions. By contrast, for the Stroop task all participants received identical instructions, and participants of both groups activated the right lateral PFC during this task, which clearly requires the control of dominant response tendencies. Nevertheless, participants in the suppression condition failed to recruit the right lateral PFC to the same extent as participants in the control condition.

It might be argued that a change in right lateral PFC activity from the first to the second task is mainly seen in the control participants, whereas the participants in the suppression group remained on the same level in the first and second task (see Figure 3). However, it is important to keep in mind that the right lateral PFC activity in the control participants reflected the ‘normal’ situation: No right lateral PFC activity during passive picture-viewing, strong right lateral PFC activity during the Stroop task. When the right lateral PFC had been activated during picture-viewing due to efforts to suppress emotions, then the subsequent activity in the right lateral PFC was reduced relative to the ‘normal’ level of activity observed in the control participants. Thus, changes in right lateral PFC activity should always be interpreted in relation to the control group.

In exploratory analyses, correlations between mean activities in the lateral prefrontal ROIs during the Stroop task with Stroop performance differed remarkably between experimental groups. In the suppression group, increased activity in the left and right lateral PFC ROIs during the Stroop task was associated with better Stroop performance, as could be intuitively expected. In the control group, this trend was reversed for the left lateral PFC ROI: More activity in this ROI was associated with *poorer* Stroop performance. This finding is puzzling for at least two reasons. First, a large research literature suggests that more prefrontal activity is associated with better performance in executive control tasks including the Stroop task [7], [26], [41], [42]. Second, the analyses are based on mean activity in the complete ROIs of considerable size (k_rLPFC_ = 279, k_lLPFC_ = 607, k_MFC_ = 129 voxels). The results of the correlational analyses are thought provoking, but should be interpreted with caution until they have been replicated in a different sample.

Although the right DLPFC has been prominently implicated in self-control efforts, we do not claim that aftereffects of self-control will consistently and exclusively affect this brain area in future investigations using different experimental manipulations and dependent variables. Due to the scarce empirical evidence to date, it is too early to make strong predictions, but based on our initial findings, we speculate that more generally those brain areas will be affected by aftereffects of self-control that are strongly involved in both self-control tasks. Recent evidence shows that beyond a common core, many self-control tasks differ in various respects [43], likely involving quite different brain areas. Thus, a combination of tasks that strongly engages, for example, the right DLPFC in one task, but not in the other, may not reveal the same pattern as in the current study. Other brain areas that have been implicated in various self-control tasks and are thus likely candidates for aftereffects of self-control exertion include, among others, the left DLPFC [44] and the right ventrolateral prefrontal cortex [45]. Thus, similar effects on the behavioral level (a first task impairing performance on the second) may be associated with different effects on the neural level (reduced activation of varying brain areas implicated in self-control across studies). The present study revealed that despite being strongly involved in both self-control tasks, an area in the MFC did not show aftereffects of prior self-control exertion. An intriguing challenge for future research will be to test the robustness of this finding and – if robust – to reveal the processes that explain why one brain area is sensitive to this kind of aftereffects of self-control exertion and another brain area is not. Taken one step further, it is even possible that the same brain area is sensitive to aftereffects under some, but not under other circumstances, depending on how psychologically effortful the respective behavioral tasks are and how much they impact on the motivation to control one’s responses.

Beyond the mere demonstration of areas affected by aftereffects of self-control exertion, it will be particularly challenging for future research to clearly identify the processes that are in turn affected by these aftereffects, such as the impaired recruitment of control to solve response conflicts. In addition, recent research suggests that lateral prefrontal areas are actively engaged in down-regulating activity in subcortical brain areas associated with impulses and craving (e.g., the ventral striatum) [46]. Thus, one crucial consequence of aftereffects of self-control may not only be the weakening of prefrontal control per se, but also the in turn enhanced activity in reward-related subcortical areas in response to a temptation, as strong activity in these areas increases the likelihood of self-control failures [7], [47].

Much of the behavioral research on the aftereffects of self-control exertion built on the assumption of a limited physiological resource that is reduced through the exertion of self-control and is later lacking for a subsequent successful self-control performance. The nature of this resource remains elusive. Some evidence suggested that the availability of glucose was one important factor [48], [49], but this assumption has been severely challenged [50]–[54]. Beyond a physiological understanding of the resource, mounting evidence underlines the important role of psychological processes such as the belief that the ability to exert control is limited, or the motivation to control [55]–[57]. For example, initial self-control efforts decrease the motivation to further exert control [58], but given appropriate incentives, individuals are well capable of counteracting deleterious aftereffects of self-control [59]. An intriguing question is how these psychological processes translate into changes in brain activity. Providing a possible explanation for the results of the present study, a reduced motivation to control may lead to less effort in subsequent self-control tasks, resulting in a decreased activation of task-relevant brain areas. Increased motivation to overcome these deficits could compensate reductions in brain activity or even recruit additional brain areas to achieve satisfactory levels of performance. These issues illustrate the great array of open questions with regard to the neural processes involved in self-control and its failures. Understanding these processes is an effort that has only just begun.
